# Iranian cancer patients’ perception of spirituality: a qualitative content analysis study

**DOI:** 10.1186/1472-6955-11-19

**Published:** 2012-10-09

**Authors:** Mozhgan Rahnama, Masoud Fallahi Khoshknab, Sadat Seyed Bagher Maddah, Fazlollah Ahmadi

**Affiliations:** 1 Nursing Department, University of Social Welfare and Rehabilitation Sciences, Tehran, IRAN; 2 Nursing Department, Faculty of Medical Sciences, Tarbiat Modares University, Tehran, IRAN

**Keywords:** Spirituality, Perception, Experience, Cancer patients, Content analysis

## Abstract

**Background:**

Spirituality is a subjective and multi-dimensional concept. The ambiguity in its meaning can create barriers in its application in both education and medicine. The present study aimed to explore the Iranian cancer patients’ perception of spirituality.

**Methods:**

A qualitative study, using the content analysis approach, was conducted. Semi-structured interviews were held with 11 cancer patients and six members of their families in one of Tehran’s hospitals and a charity institute. The data generated were transcribed verbatim and content analysis approach was used for data reduction, naming data, obtaining analytical code and determining categories and themes.

**Results:**

Three themes (and seven sub-themes) emerged from the data analysis: 1) God as the spiritual truth (relationship with God and trust in God), 2) Moralities as a spiritual sign (considering personal and social moral codes) and 3) Spiritual resources as the source of hope (religious, personal and social resources).

**Conclusions:**

Overall, in the view of cancer patients, spirituality can be defined in a religious context. However, some of them believe in morality beside religiosity, so health care staff must pay due attention to these aspects, to provide them with the opportunity to use spiritual resources.

## Background

Cancer patients are prone to spiritual distress upon facing with their diagnosis, change in disease stage and the difficulties of ending their lives [1]. This is because they suffer from lack of meaning, value and purpose in their lives due to the severe physical and functional damages accompanying the disease [2]. According to a survey by the New York Cancer Center, cancer patients and their families should be taken care of in an absolutely safe and secure setting so that they can freely express their physical, emotional and spiritual needs [3]. As Schulz notes, cancer patients’ spiritual needs often include finding meaning and hope, having access to spiritual resources and extrapolating meaning from the pain and suffering [4]. Essentially, in the case of a life crisis, spirituality arises as a serious issue for both patients and their families [5], since spirituality is a dimension through which cancer patients can fight the sense of fear and loneliness throughout their disease [6].

However, anyone may have his or her own interpretation of the concept of spirituality. Also the person’s age, sex, race, culture, previous personal experiences, and the stage of life, which the person is experiencing, affect the expression of his spirituality [7]. There is still is no unified definition of spirituality, which can direct the research and treatment of cancer [6], because spirituality is a multifunctional concept [8] and no comprehensive definition that captures its entirety has been offered so far. Among the definitions are: "One’s innate nature" [8,9], "innate desire for meaning-making" [10], "The feeling of attachment to God or a superior power" [9] and "provider of meaning and hope in life" [8,9,11]. In addition, the ongoing discussions regarding spirituality and religion have further complicated the topic of spirituality. Religion is defined as an organized system of beliefs, activities, rituals and symbols, which facilitate the sense of attachment to God, a superior being, an absolute power or a designed truth [12].

From the viewpoint of Muslims, there exists no difference between religion and spirituality, and spirituality is essentially interwoven with religious thoughts and activities [13]. However, in the western world, spirituality is not synonymous with religion [14]. To westerners, spirituality is a more comprehensive concept than religion that embraces philosophical thoughts on life, meaning and purpose [15].

Perhaps the best definition of spirituality can be expressed as follows: " it is a way through which human beings recognize the exalted meaning and value of their lives." In their quest for this meaning and value, many people have turned to religion. However, some others seek comfort in spiritual concepts outside the realm of organized religion [16].

Spirituality is associated with both culture and religion and all of these three factors influence our understanding of health and disease. Thus, in clinical environments, there must be a deep understanding of religious/spiritual concepts. Similarly, confusion between the two realms of religion and spirituality must be avoided [6]. Establishing a clinical care system that takes into account all of the patients’ spiritual needs is one of the most crucial responsibilities of nurses [11]. However, in busy hospital environments, nurses might overlook the patients’ spiritual needs [17]. According to Grant (2004): "Health experts have discovered the significance and effect of spiritual dimensions, and there is a consensus that spiritual needs must be catered for and satisfied. However, most of them feel that there is not enough time and professionalism devoted to spiritual care" [18]. Mooney (2007) too considers the existing problems concerning a direct definition of spirituality, the lack of true understanding of nurses regarding this issue and the lack of understanding of the significance of discussing the issue the main reasons for the failure of some nurses to attend to the spiritual needs of patients in clinical treatment [11].

Accordingly it can be concluded that there is a certain ambiguity in understanding the concept of spirituality. Keeping in mind the fact that the experiences of patients and their families can play an underlying role in understanding this concept, and that the viewpoints and experiences of cancer patients regarding spirituality in Iran have not been studied, a better understanding of this issue in Iran is required. In particular, the effect of culture and personal codes of behavior in understanding spirituality means that Iranian patients’ interpretations of spirituality may not necessarily be similar to those of other cultures. Thus this study has been designed to evaluate the Iranian cancer patients’ perception of spirituality.

## Methods

This qualitative study was done with the purpose of exploring the Iranian cancer patients' perception of spirituality as well as evaluating the patients’ experiences about their spiritual needs during the nursing care services using a conventional content analysis approach.

### Data collection

The data were gathered through semi-structured interviews. First, some general questions were proposed to start the interviews such as: "Please define spirituality according to your view." and "What is your description of spiritual activities?" The trend of the interviews was based on the participants’ answers. Then a couple of questions about the patients’ experiences of the offered nursing cares during their hospitalisation were asked. The time and location of the interviews were arranged by agreement with the participants. The length of each interview varied from 45 to 120 minutes.

### Participants

The population of this study consisted of 11 cancer patients in the hospitals and 6 members of their families in one of Tehran’s [1] hospitals and Behnam Daheshpour Charity Institute, who were selected through purposive sampling. To consider sampling with maximum diversity, the participants were chosen from a wide range of people with different characteristics (age, gender, socioeconomic status, disease phase, etc.).

### Data analysis

All interviews were conducted, recorded, verbatim typed, reviewed, coded and immediately analysed by the first researcher. A content analysis approach was used for data analysis. According to the content analysis process, at first, each interview was read several times carefully to gain a universal and primary perception, and then important statements were underlined (to identify the initial codes or meaning units that exist in the interview text about the participants’ perception of spirituality). In the next phase, these similar meaning units were abstracted for transparency of the meanings and labelled in the form of themes and sub-themes. Indeed, data analysis was done in a constant and concurrent way along with the data collection. The data collection process was continued until data saturation – when adding further data showed no new information and the extra collected data were redundant. Finally, the main themes were extracted.

### Rigour

In this research, validity and reliability of the study were examined using the criteria suggested by Guba and Lincoln [19]. To establish credibility, the author had sufficient cooperation and interaction with the participants. Reviews were carried out by the external supervisors, and the professors’ additional comments were also used. The researchers checked the dependability of the data through performing activities such as consulting the supervisor, associate professors and experts to review the material. Confirmabilty was conducted by setting aside all presumptions and prejudices. Besides, the findings’ validity was also confirmed by the participants.

### Ethical consideration

This study was conducted after getting the approval of the Ethics Committee of Tehran University of Social Welfare and Rehabilitation Sciences and obtaining written permission from this university. In this research, the participants were asked to sign the informed participation form. They were also assured that their private information would be kept secret when the results were published. Furthermore, they were emphatically informed that they could stop their cooperation with the researcher at any step of the study this wished.

## Results

The participants of this study were 11 cancer patients and 6 members of their families who had come to one of Tehran’s hospitals and Behnam Daheshpour Charity Center. The patients’ age varied between 27 and 65 while the age of their family members was between 24 and 60. The patients consisted of 6 females and 5 males, but all of their family members participated in the study were female. The patients’ cancer types were breast cancer, intestinal cancer, liver cancer, spinal cord tumor, brain tumor, lung cancer and testicular cancer. The study’s main objective was to discover the patients’ perception of spirituality. The findings of this research assisted the researchers to detect the following three main themes: 1 – God as the spiritual truth, 2 – Moralities as a spiritual sign and 3 – Spiritual resources as the source of hope.

### Theme 1: God as the spiritual truth

To understand the participants’ perception of the meaning of spirituality, they were asked to define spirituality in their own words and describe spiritual activities. Subsequently, it was found that all of them believe in the religious aspect of spirituality and, to define spirituality, they used such terms as "relationship with God" and "trust in God".

### Relationship with God

When defining spirituality, a large number of the participants mentioned "relationship with God", and religious activities including "saying prayers ( Namaz )" [2] , "prayer", "visiting the shrines and holy places", "mentioning God", "Fasting" and "reciting" the Holy Quran" as important parts of spiritual practice, in their opinion. For instance, participants 8, 3 and 6 made the following points: 

'Spirituality can be noticed from several perspectives but they all share in one point: Joining to the creator of lights, love, cure, beauty and all good things.' (Participant 8, 60 years old family member)

'Saying prayers ( Namaz ), visiting holy shrines, praising God and reciting the Holy Quran are among spiritual activities.' (Participant 3, 37 years old male patient)

'I relate to God through Saying prayers ( Namaz ) and blessing, however, now as a patient, I mainly used to constantly bless.' (Participant 6, 33 years old male patient)

Some participants also addressed "inner relationship with God" and "talking with God" as spiritual activities: 

'I have an inner relationship with God; whenever I am in contact with him, my wishes come true. So I always talk to him, no matter if it is day or night.' (Participant 5, 28 years old female patient)

'I just talk to God. I neither say prayers nor do fast. But I always thank Him when I go to bed.' (Participant 7, 57 years old female patient)

### Trust in God

Some participants mentioned "trust in God" and "doing His orders" as their definition of spirituality: 

'Spirituality means having trust in God and restoring to Imams (The Prophet’s Household) [3] .' (Participant 3, 37 years old male patient)

'Spirituality means doing the orders of God.' (Participant 12, 44 years old family member)

### Theme 2: Moralities as a spiritual sign

Some participants added moral aspects besides religious aspects in the defining of spirituality. In their opinion, ethical considerations in personal and interpersonal fields as well as religious practice as indications of spirituality.

### Personal ethical consideration

In the opinion of some participants, being trustworthy, having no hatred towards others, having an appropriate appearance, nice manners and honesty in speech and practice, lacking hypocrisy, and not being in the boundaries of material subjects are among the sings of spirituality: 

'In my idea, a religious man avoids telling lies or deceiving others. He does not envy others’ belongings, does not violate others’ rights, and listens to God’s orders. Of course being religious is not just being careful about the appearance and growing the beard and so on. A spiritual man may not have a spiritual appearance unless his beliefs are religious too.' (Participant 3, 37 years old male patient)

'Spirituality means that you can be a good person and do good deeds to other people, then you can also expect good things from others.' (Participant 5, 28 years old female patient)

'I don’t think spiritual individuals have a particular appearance or face. Actually, some people are called the "salt of the earth". I think you can reach to this level if your soul is clean. If you don’t have the feeling of hatred, then you can easily forgive others, no matter what has happened.' (Participant 6, 33 years old male patient)

### Social ethical consideration

In other participants’ opinion, respect for others’ rights, offering love to others, not deceiving others, being polite and respectful in relationships, offering help and support to others, and obeying the do’s and do not’s of God (Halal and Haram) can be defined as spirituality: 

'Spiritual activities include such acts as offering help and support to others. For example, once someone is in trouble, you can talk to him/her and relieve his/her pain and grief.' (Participant 5, 28 years old female patient)

'Spirituality is found in the sincere work. All of these contributors who comes here and offer biscuits and juice to the patients’ accompaniers are spiritual individuals. These are valuable deeds and are beyond the definitions I previously knew about spirituality. Before this, I supposed that spirituality is only in religion.' (Participant 6, 33 years old male patient)

'The basic component of spirituality is to love all human beings; as human needs to be first treated and then provided with spiritual needs, which initiate from loving human beings in line with loving God.' (Participant 8, 60 years old family member)

### Theme 3: Spiritual resources as the source of hope

Finally, the participants were asked to mention what spiritual resources they usually use. Their answers revealed that the spiritual resources most used by them were "worship activities", "seeking for religious appeal" and "religious beliefs". But some participants addressed individual and social resources beside religious resources as spiritual recourses.

### Religious resources

#### Worship activities

The interviews with the participants showed that the common forms of worship such as saying prayers (Namaz), fasting, Quran citation, individual prayers and other individuals prayer for a person are considered as spiritual resources for them: 

'Once people do their religious duties such as Namaz and fasting, their position will be gradually evolved and they will be promoted spiritually.' (Participant 2, 36 years old female patient)

'In my opinion, everyone must see what has been told by the source and root of spirituality. One might be Muslim, Hindu or Buddha; I don’t say everyone should be like me. I think everyone must see what he must do according to the orders of his/her spirituality source and do everything that pleases that source. We as Muslim have the Holy Quran as our law and rules book. We must think about God and see what He has ordered us in this book. Well, saying daily prayers is very important.' (Participant 12, 44 years old family member)

#### Seeking for religious appeal

Through interviewing the participants, it was found that reliance on some spiritual forces such as trust in God, restoring to Imams, visiting the shrines and holy places and making in vow are regarded as a spiritual resource for some of them: 

'The first thing that came to my mind the day my child’s disease was in its most critical phase and they wanted to do a bonemarrow biopsy and I was wondering and worrying what would happen then and what would be the diagnosis . Everything was shaky. I suddenly called my brother and told him to take me to the village of Boumehen (Suborbs of Tehran). There he sacrificed a sheep. I think making sacrifice (Killing a sheep) for charity seeking for the God’ satisfaction is very good in these situations.' (Participant 12, 44 years old family member)

'In my idea, these types of sick persons have a great deal of tolerance. At least I myself was very patient. Whenever someone called me and asked about my condition, I used to answer that I was good instead of saying I am about to die! I used to say: ‘Today I am better than yesterday’. I had no choice except having trust in God and patience.' (Participant 13, 45 years old female patient)

#### Religious beliefs

The interviews revealed that some of the participants of this study use a number of religious beliefs as their spiritual sources: 

"Believe in the possibility of gaining health from God", "believe in miracles", "believe that this is God defines the length of one’s life" and "patient’s believe in the efficiency of holy components and materials".

For instance, participants 5, 13, 14 and 15 made the following points: 

'Everyone has some kind of beliefs in his own God. I had this belief from the past since I used to tell those in trouble to have faith in God. I swear He gives us everything you may want. Now I am asking for my health and I am sure He will give me that.' (Participant 5, 28 years old female patient)

'You know, on some occasions, you think no one can help you anymore. It is better to rely on a greater power. For instance, I used to take some pills made in Switzerland, which are very good for nausea. Everyone used to tell me they work wonders, they do miracles, but they did nothing for me, I think the only thing that made a miracle to me was my faith in God, which brings me peace. Once I am calm, I can tolerate my physical problems more easily.' (Participant 13, 45 years old female patient)

'After I was hospitalised and undergone a surgery, I got better. I saw there were a lot of people like me and I am not the only one; this made me feel better. Indeed, you have to get along with this. It is God’s will.' (Participant 15, 49 years old family member)

'If my fate is to continue my life in this world, then I will be cured If not, I don’t regret at all because I have done everything I could, I trust in God only.' (Participant 14, 43 years old male patient)

### Personal resources

Interviews with the participants revealed that, while dealing with the disease, the individual’s inner forces and beliefs can be of a great assistance: 

'I basically believe that everything that happens to us is our fate. For instance, my death date is a predefined matter, so there is no use in struggling futilely.' (Participant 9, 58 years old female patient)

'Once I came to the doctor’s office to show my tests results, and wanted him to remove my stitches, I asked him if my cancer was benign or malignant. He asked me: "Whoever accompanies you, tell them to come in". My husband and my mother were there. Once I saw their talk took longer than expected, I found that my guesses were true. But it was as if some inner forces told me: "be patient, don’t surrender.' (Participant 2, 36 years old female patient)

### Social resources

Through the interviews, it was discovered that one of the helpful resources for the patients is their relationship with their family members, spouse, friends, and even the health care staff: 

'My son had told his wife: May family has a great influence on my morale. I didn’t lose my heart because my mom and others treated me very well'. (Participant 12, 44 years old family member)

'My kids have grown. My sons are now adults. You know, my husband is a farmer and does not know anywhere, so my sons usually bring me to Tehran. I am totally pleased with my sons. They support me both financially and emotionally. They give me hope.' (Participant 15, 49 years old family member)

'I swear that I had some guesses about my diseases, so I was afraid to go to the doctor. Maybe this was my factor that made me visit the doctor this much late. Finally, believe it or not, I visited the doctor after my wife made an appointment.' (Participant 6, 33 years old male patient)

'Now, I visit my surgeon once every 6 months. I adore him very much. I remember once he came near my bed after the surgery and told me: "I have named your operation as a new birth". Well, since then, every year I hold a ceremony for my birthday in addition to my real birthday. It is very important to see how the doctor treats his patients.' (Participant 7, 57 years old female patient)

## Discussion

In this study, all participants put the emphasis on the religious aspects of spirituality and considered activities such as saying prayers (Namaz), prayer, visiting holy places, etc. as spiritual activities, and only a few of them mentioned moral aspects together with religious aspects. They believed that having individual and social moralities is a sort of spiritual activity. A comparison between the findings of this study with those of some other studies revealed some similarities and differences as follows: 

"In the present study, relationship with God, having faith and trust in God and obeying God’s orders were defined as spirituality in the view point of the participants. In a study conducted by Schulz (2008), the participants defined spirituality as a deep relationship with a superior power (God or the Holy Spirit), faith, synonymous to religious beliefs, and something comprising immortality and eternity [4]. Although, in terms of emphasising the religious aspect of spirituality, the findings of their study are consistent with ours. The participants in their research did not mention the moral aspects of spirituality. In another study, conducted by Penman et al. (2009), the participants defined spirituality as believe in God, relationship with others and religion [5]; although this study, like ours, mentions religion and relationship with others. However, none of other items extracted in our study, such as "being good", "love", "having a clean heart", "charity" and "being fair", have been mentioned in that study. Wong (2004) also specified that religion includes official and organised religious beliefs and ceremonies. Even though many people express their spirituality through religion, yet some of them show it by "coherence", "happiness", "calmness", "love", "awareness" and "meaning in life" [9]. Many of the concepts introduced by Wong’s study were not mentioned in our study and the only similarities between these two studies were "religion" and "love". On the other hand, comparison of the present study with some other studies shows completely different results. For instance, Puchalski and Romer define spirituality as something that enables an individual to have the experience of metaphysical meaning in life. In addition, Karasu and Brady believe that spirituality consists of two components: faith and meaning. They mean by faith to believe in any metaphysical power and not necessarily in God [20]. However, in our study, the participants mentioned relationship with God and put the emphasis on having trust in or relying on God and obeying His orders. It is clear that the comparison between theses two studies implies significant differences in the conception of spirituality between our religious society – where religion is coupled with people’s culture – and some other societies; as in our society, there is a strong emphasis on the religious aspect of spirituality. In this regard, the study of Hyman (2006) conducted through interviews with experts of different religions about the meaning of religion and spirituality, showed that 83% of the participants believed that religion and spirituality are two concepts that overlap each other, and only 13% of them (who were mainly Muslims) considered religion and spirituality as the same [21]. Cheraghi (2009) mentions the following: "A large number of researchers, especially in the western societies, accept that religion and spirituality are different but related. For some people, being religious is an external expression of inner spirituality, and for others, it includes philosophical ideas about the meaning and purpose of life. There is no agreement on a unique definition about the spirituality." [13] Mahmoodishan (2010) states that spirituality is a subjective concept, extremely subjective. It is multidimensional and there is no agreement on its definition [22].

Based on the results of this study, relationship with God through religious practices, inner relationship with God and talking to him, and relationship with the self and others are recognised as spiritual practices. In the study of Penman et al. (2009), factors such as maintaining relationships (in the form of intimacy, showing concern, presence, offering one’s services and showing attention and support), love (as lack of selfishness and belonging to others), and participation in religious activities (like saying prayers, which is regarded as talking to God) were considered as the participants’ spiritual challenges [5]. This is similar to the present study considering such items as "talking with God", "doing religious activities", "having a deep relationship with God" and "offering support and love to others.

In the opinion of the participants in the study of Alcorn, spiritual and religious activities included: blessing, religious services, meditation and study of religious texts [23]. His research is similar to the present study with regard to the items such as "prayer", "religious activities" and "study of religious books"; however, meditation has not been mentioned by the participants of our study. Comparison of the present study with some other researches also indicates different results. For instance, in a research conducted by Lopez, most of the participants listed activities such as family issues, sport, listening to music and relationship with others as religious activities, and a few of them referred to yoga and meditation [24]. This study is similar to our study only in terms of its focus on “relationship with others”, while in many aspects; it is different from the present study (i.e. doing sports, playing music, practicing yoga and meditation). It seems that the cultural and religious differences are effective to explain such dissimilarities.

Our study found that the religious resources used by the patients include worship practices (saying prayers, fasting, reciting the Holy Quran, individual blessings and others’ blessing for the individual) and religious appealing (through having faith in God and relying on Imams (The prophet Mohammad’s Household), sacrifice and visiting the holy places). Dehghani found that Quran recitation can serve as an effective tool to improve and enhance the spiritual health in chemotherapy patients [25]. Taleghani (2005) also suggested that an important factor in female breast cancer patients in the prognosis phase is their reliance on charity, visiting the holy places and reliance on Imams [26]. Besides, Aquino et al. (2007) reported binding to the religion and seeking for empathy from individuals or groups act as a rescue path for cancer patients [27]. These are all in agreement with the results of the current study.

Religious beliefs about the possibility of improvement by God’s will and miracles are also among the religious resources – making the flame of hope of survival in their heart – as mentioned by the participants of this study. Aquino et al. (2007) claimed that all patients have the hope of finding a new chance for living by the belief that God can control even the worst situations [27]. In this regard, Surbone (2009) asserts that many cancer patients, goaded by the ambiguity in their current situation and uncertainty of their future, rely on religious beliefs – as a power and hope source – and can cope with their fear and loneliness during their illness [6]. This claim is also consistent with the results of the present study. Astonishingly, Puchalski 2009 states that spiritual and religious beliefs can aggravate the illness through the creation of emotional distress [28].

In this research, individual and social resources suggested as a source of hope resource by the participants include individual characteristics and beliefs and connection to the family members, spouse, friends, health staff and other patients. Similarity, Chiu found that the participants believed that family relationships, national-cultural values, religion, alternative treatments (such as sport, meditation, using medical plants and some nutritive programs, as well as making use of the natural environment), creative activities (art works and writing) and support groups (such as the Breast Cancer Support Association) are among the factors for gaining spiritual power [29]. However, they mentioned some other factors such as alternative treatments, creative activities and support groups, which are not found in the present study. These findings show that seemingly cultural differences are effective in obtaining different results.

## Conclusion

In general, the experiences of the participants of this research indicated that all of them put an emphasis on the religious aspect of spirituality; however, since some of them believed in the moral aspects of spirituality, it seems compulsory that health caregivers to provide the patients with the opportunity for performing spiritual activities in both aspects. Furthermore, since using spiritual resources seems necessary to perform these practices, health caregivers can assist the patients in their spiritual practices through providing the needed conditions and opportunities for using these resources and solving their problems. Also it is recommended to make consultations with the professionals to recognise and enhance the inner forces through increasing and strengthening family relationships, which were among the spiritual sources proposed by the participants.

The researchers of this study also highlight that this research has been conducted on Muslim Iranian patients and cannot be generalised for the patients of other religions or countries. It is worth noting that the experiences of cancer patients have been generally explored, and there was no emphasis on a particular type of cancer. In addition, it is recommended to perform similar studies on a particular type of cancer and on the patients from other religions.

## Endnotes

^a^Based on the agreement made with the authorities of this hospital, it was decided to keep its name secret until the final results achieved and the patient’s permission is obtained.

^b^An organized form of relationship with God.

^c^The twelve holy grandsons of the prophet of Islam (peace be upon him).

## Competing interests

The authors declare that they have no competing interest.

## Authors’ contributions

MR, MFK and SSBM designed the study; MR collected data; MR, MFK, SSBM and FA contributed to data analysis, interpretation of findings and drafting of the manuscript. Both authors read and approved the final manuscript.

## Pre-publication history

The pre-publication history for this paper can be accessed here:

http://www.biomedcentral.com/1472-6955/11/19/prepub
